# *Rickettsia*-Host-Tick Interactions: Knowledge Advances and Gaps

**DOI:** 10.1128/iai.00621-21

**Published:** 2022-08-22

**Authors:** Hwan Keun Kim

**Affiliations:** a Center for Infectious Diseases, Department of Microbiology and Immunology, Stony Brook University, Stony Brook, New York, USA; University of California, Santa Cruz

**Keywords:** *Rickettsia*, tick, spotted fever, rickettsiosis, pathogenesis, endothelial cell, macrophages

## Abstract

Ticks are hematophagous ectoparasites capable of transmitting multiple human pathogens. Environmental changes have supported the expansion of ticks into new geographical areas that have become the epicenters of tick-borne diseases (TBDs). The spotted fever group (SFG) of *Rickettsia* frequently infects ticks and causes tick-transmitted rickettsioses in areas of endemicity where ixodid ticks support host transmission during blood feeding. Ticks also serve as a reservoir for SFG *Rickettsia*. Among the members of SFG *Rickettsia*, R. rickettsii causes Rocky Mountain spotted fever (RMSF), the most lethal TBD in the United States. Cases of RMSF have been reported for over a century in association with several species of ticks in the United States. However, the isolation of R. rickettsii from ticks has decreased, and recent serological and epidemiological studies suggest that novel species of SFG *Rickettsia* are responsible for the increased number of cases of RMSF-like rickettsioses in the United States. Recent analyses of rickettsial genomes and advances in genetic and molecular studies of *Rickettsia* provided insights into the biology of *Rickettsia* with the identification of conserved and unique putative virulence genes involved in the rickettsial life cycle. Thus, understanding *Rickettsia*-host-tick interactions mediating successful disease transmission and pathogenesis for SFG rickettsiae remains an active area of research. This review summarizes recent advances in understanding how SFG *Rickettsia* species coopt and manipulate ticks and mammalian hosts to cause rickettsioses, with a particular emphasis on newly described or emerging SFG *Rickettsia* species.

## EVOLUTION OF *RICKETTSIA*

Rickettsiae (alphaproteobacteria; *Rickettsiales*, *Rickettsiaceae*) are small (0.3- to 0.5- by 0.8- to 2.0-μm) Gram-negative bacteria with an obligate intracellular life cycle circulating between mammalian hosts and hematophagous arthropod vectors (e.g., ticks, mites, fleas, and lice) in nature. Early studies using electron microscopy identified intracellular *Rickettsia* with a trilaminar cell membrane surrounded by a slime layer (1). The rickettsial outer membrane is decorated with lipopolysaccharides that are highly immunogenic and responsible for cross-reactive Weil-Felix antibodies (2). On the basis of the genome sequence, antigenic properties, and disease attributes, rickettsiae are categorized as belonging to the spotted fever group (SFG), typhus group (TG), transitional group (TRG), and ancestral group (AG) (Table 1) (3, 4). Rickettsiae are transmitted to mammalian hosts during blood feeding by infected ticks and mites or by contaminated feces of infected lice and fleas. Humans do not contribute to rickettsial circulation in nature, except for Rickettsia prowazekii, for which they serve as a reservoir and suffer from recurrent Brill-Zinsser disease (5, 6). Comparative and phylogenomic analyses identified that while adapting to an intracellular life cycle, the chromosomes of *Rickettsia* evolved via progressive reduction, resulting in small genomes ranging from 1.1 to 1.5 Mbp (encoding ~800 to 1,300 proteins) with predictions of ~700 core genes, an ~30% G+C content, and a coding capacity of 69 to 84% (7). Through reductive genome evolution, *Rickettsia* lost genes involved in metabolic pathways and has a limited ability to synthesize amino acids and nucleotides, mimicking symbiotic bacteria (8). To compensate for gene loss, *Rickettsia* species developed parasitic mechanisms whereby a large array of transport systems pilfers essential metabolites for their survival and replication within the host cytosolic compartment (8). Thus, identifying specific metabolic pathways missing in *Rickettsia* may provide critical knowledge in gaps in developing an axenic medium that supports rickettsial extracellular replication and novel therapeutics that target essential transport mechanisms.

**TABLE 1:** *Rickettsia* groups and diseases

Group	Species	Disease	Vector
Spotted fever^*a*^	R. rickettsii	Rocky Mountain spotted fever	Tick
	R. conorii	Mediterranean spotted fever	Tick
	*R. parkeri*	*R. parkeri* rickettsiosis	Tick
	*R. philipii* (*Rickettsia* sp. 364D)	Pacific Coast tick fever	Tick
	*R. africae*	African tick bite fever	Tick
	*R. japonica*	Japanese spotted fever	Tick
	*R. heilongjiangensis*	Far-Eastern spotted fever	Tick
	*R. honei*	Flinders Island spotted fever	Tick
	*R. amblyommatis* ^*b*^	Mild spotted fever	Tick

Typhus	*R. prowazekii*	Epidemic typhus	Louse
	R. typhi	Murine typhus	Flea

Transitional^*a*^	*R. felis*	Flea-borne spotted fever	Flea
	*R. akari*	Rickettsialpox	Mite

Ancestral	*R. bellii*	Nonpathogenic	Tick
	*R. canadensis*	Nonpathogenic	Tick

a A nonexhaustive list.

b Rickettsiae presumptively associated with human diseases.

Rickettsiae have evolved to adapt to diverse environmental conditions, including various arthropod vectors and mammalian hosts, and display various degrees of mutualism and pathogenicity. For instance, several R. felis (TRG) strains have been identified, sequenced, and characterized for their diverse genetic makeup, pathogenicity, and vector adaptations (9). *R. felis* in booklouse (Liposcelis bostrychophila) is involved in the development of oocytes, maintained strictly via transovarial transmission, and is considered nonpathogenic in mammalian hosts (10). In contrast, flea-borne *R. felis* is responsible for many febrile diseases of unknown origins in areas of endemicity (11–14). Comparative genome sequence analysis identified genomic sites that are conserved and divergent between flea-derived and booklice-derived *R. felis* strains, suggesting that genetic variability may contribute to vector specificity and virulence in mammalian hosts (15). However, further studies are required to investigate the unique genetic traits of *R. felis*, its adaptation to different arthropod vectors, and their relationship to virulence in mammalian hosts.

Interestingly, *R. prowazekii* (TG), which has the smallest genome, causes the most severe and lethal disease (epidemic typhus), which has claimed countless lives over the last centuries (16). This paradoxical inverse correlation where increased pathogenicity is associated with genome reduction has also been described for other pathogenic bacterial species such as Mycobacterium leprae, Yersinia pestis, and Streptococcus suis (17–19). While we do not fully understand the complex evolutionary processes of rickettsial gene deterioration, comparative and whole-genome sequencing analyses suggest that the increased pathogenicity of *Rickettsia* is not associated with novel virulence gene acquisition but instead is correlated with efficient and/or reduced gene regulation in virulent *Rickettsia* species (16, 20–26). Despite ongoing reductive genome evolution, similar studies identified various degrees of conservation and expansion of genes encoding tetratricopeptide repeats, ankyrin repeats, toxin-antitoxin modules, stress response regulators (SpoT), ADP-ATP translocases, proteins involved in the type IV secretion system (T4SS), surface cell antigens (Sca), hemolysins, phospholipases, and uncharacterized proteins with putative virulence functions (27–31). Gene rearrangements, deletions, and mutations have been implicated in the attenuated virulence of *Rickettsia*, but additional studies are needed to determine the functional significance of genetic variants (32–34). The information gained from comparing the genomes of *Rickettsia* strains has provided significant insights into the gene conservation, divergence, and evolution of *Rickettsia* and enabled investigators to identify putative virulence genes important for the rickettsial intracellular life cycle in mammalian hosts and arthropod vectors and to correlate these with diverse pathogenic mechanisms subverting host immunity. However, the selective pressure and molecular mechanisms enabling *Rickettsia* species and strains to maintain and reduce their genome sizes remain unknown.

## SPOTTED FEVER GROUP *RICKETTSIA*

Ticks are hematophagous ectoparasites capable of transmitting multiple human pathogens of public health importance. Recent environmental changes have contributed to the expansion and invasion of ticks into new geographical areas that have become the epicenters of tick-borne diseases (TBDs) (35, 36). Ticks require blood meals for their continued development, reproduction, and survival. The SFG rickettsiae infect ticks and cause tick-transmitted rickettsioses in areas of endemicity where ixodid ticks support host transmission through their bites during blood feeding. Infected ticks become a primary reservoir of SFG *Rickettsia* species, providing a lifelong opportunity to transmit and amplify these pathogens in mammalian hosts (Table 1). In North and South America, Dermacentor variabilis, D. andersoni, Rhipicephalus sanguineus, and Amblyomma sculptum are confirmed vectors of R. rickettsii. In addition, A. maculatum, A. tigrinum, and A. triste transmit R. parkeri rickettsioses. In Europe and the Mediterranean littoral to India and Africa, R. sanguineus is the most common vector for R. conorii. R. africae has been associated with several tick species of the genus *Amblyomma* in Africa. In Asia, R. japonica has been frequently isolated from several tick species that belong to the genera *Haemaphysalis*, *Ixodes*, and *Dermacentor*. With the advances in molecular genetics in the past decades, several novel SFG rickettsiae have been identified and characterized for their association with tick reservoirs and contributions to numerous tick-borne rickettsioses throughout the world, for instance, R. heilongjiangensis in Dysmicoccus sylvarum and *Haemaphysalis* ticks and R. honei in Bothriocroton hydrosauri, Haemaphysalis novaeguineae, and *Ixodes* species (37–39). These epidemiological data strongly advocate for the importance of tick surveys in preventing and managing tick-borne rickettsioses and understanding the pathophysiology of *Rickettsia* in tick and host transmission. Antibiotic treatment with doxycycline is most effective when initiated early in the course of tick-borne rickettsioses (40). Delayed diagnosis and antibiotic treatment are associated with adverse clinical outcomes such as increased rates of hospitalization, admission to an intensive care unit, a delayed time to recovery with complications, and mortality (40–42). Increased levels of *Rickettsia*-specific immune titers represent serologic confirmation of rickettsial infections; however, nonspecific clinical symptoms (for instance, fever, headache, myalgias, and nausea) and limited access to molecular diagnostic tools in reference laboratories prohibit the prompt diagnosis and treatment of rickettsial infections. As a result, the actual incidence of tick-borne rickettsioses is predicted to be much higher, and the case fatality rate of SFG rickettsioses remains high in many parts of the world (43–45). Overall, the public health burden of tick-borne rickettsioses remains significantly underestimated.

## TICK TRANSMISSION OF SFG *RICKETTSIA*

**FIG 1:**
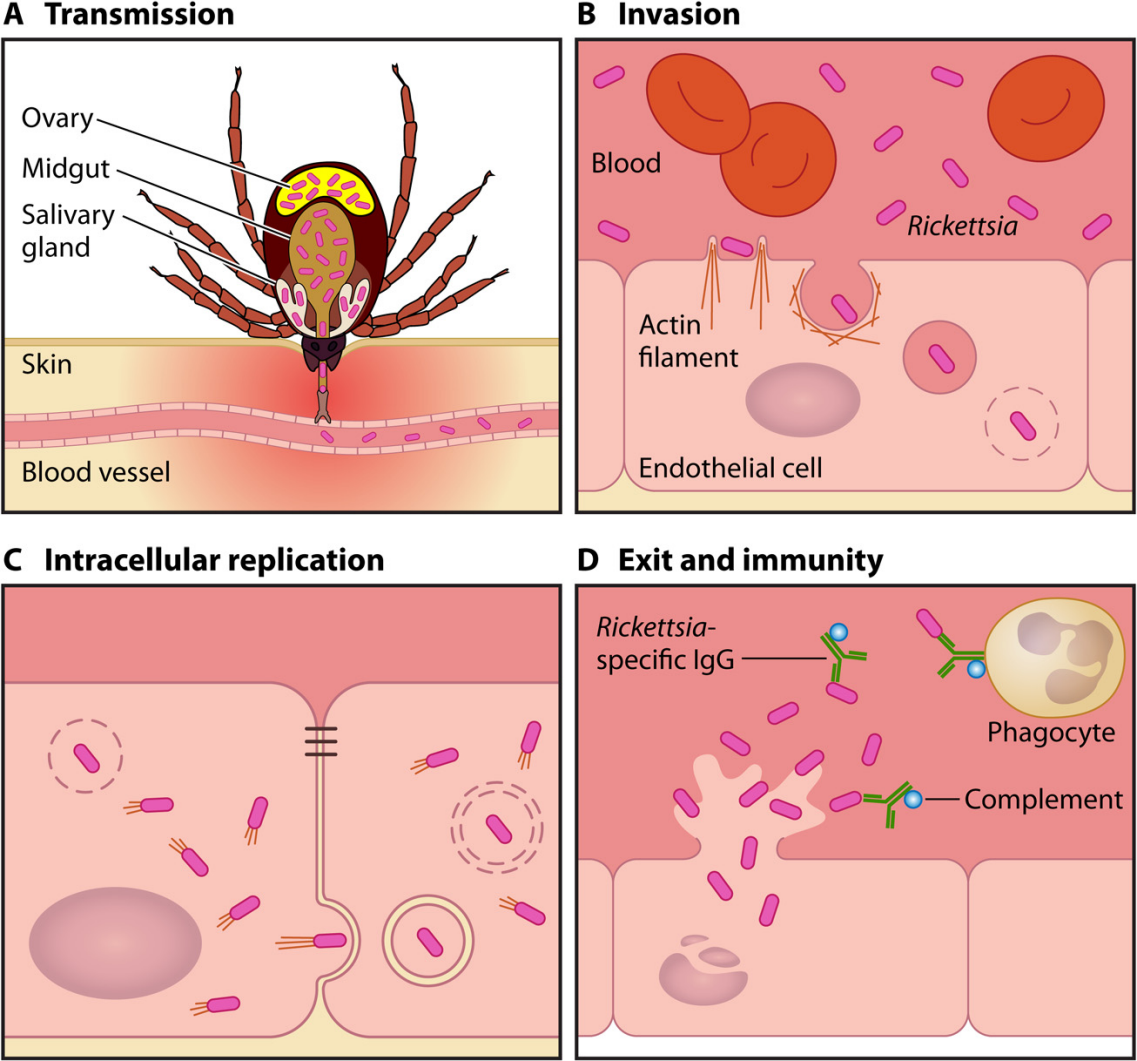
Life cycle of tick-borne *Rickettsia*. (A) Spotted fever group rickettsiae infect salivary glands, midguts, or ovaries of susceptible ticks. Infected ticks transmit rickettsiae through their bites during blood feeding, along with immunomodulatory components in tick saliva. (B) Within the bloodstream, rickettsiae target vascular endothelial cells, inducing actin-mediated uptake and the subsequent release of rickettsiae into the cytoplasm. (C) The intracellular replication of rickettsiae requires multiple virulence factors for immune evasion, host cell invasion, membrane lysis, and nutrient uptake. (D) Rickettsiae destroy vascular endothelial cells, causing local and systemic vasculitis. Survival of rickettsiae within phagocytes is essential for clinical disease. Infected individuals elicit *Rickettsia*-specific antibodies and T-cell responses for immune protection.

During blood feeding by infected ticks, rickettsiae in tick saliva are introduced into the dermis and small capillaries, seeding initial infection with varying degrees of local inflammation and cellular infiltrates such as macrophages (Fig. 1) (46–50). The underlying molecular mechanisms mediating the initial acute phase of tick-borne rickettsiosis in humans are largely unknown, as many patients seek medical interventions several days after the onset of clinical symptoms. However, several factors contribute to the successful transmission of SFG *Rickettsia*, for instance, the duration of tick attachment, bacterial loads in tick saliva, and the transmission efficiency of *Rickettsia*. Under laboratory conditions, R. rickettsii transmission occurred as soon as 8 h after D. variabilis bites on guinea pigs, and the severity of clinical disease was dependent on the duration of tick attachment (51). This study corroborates reported cases of *R. parkeri* rickettsiosis in patients with <8 h of tick attachment (49, 50). The bacterial loads in tick saliva and salivary glands and the capacity to transmit *R. parkeri* by infected A. maculatum ticks played significant roles in successful *R. parkeri* transmission and causing local inflammation at the tick attachment sites on Sprague-Dawley rats (52). Tick saliva contains an arsenal of multiple immunomodulatory agents that affect host hemostasis and immune defense mechanisms (53, 54). Besides facilitating tick attachment and blood feeding, the immunomodulatory properties of tick saliva contribute to the enhanced transmission of several tick-borne pathogens, including SFG *Rickettsia* (53–55). Skin-resident dendritic cells (DCs) sense the local inflammatory environment and regulate tissue homeostasis, immune tolerance, and T-cell responses against invading pathogens (56–59). A recent study reported that prostaglandin E_2_ (PGE_2_) in A. sculptum saliva dampened the proinflammatory immune responses of DCs infected with R. rickettsii (60). The abundance of PGE_2_ increased as the ticks continued to blood feed, potentially assisting in the survival and hematogenous dissemination of *Rickettsia* (60). Indian rhesus macaques (Macaca mulatta) exposed to *A. maculatum* adult ticks and subsequently infected with an intradermal injection of 10^7^ *R. parkeri* cells suffered from persistent *R. parkeri* infection at the inoculation site (eschar) and systemic dissemination of *R. parkeri*, suggesting that tick feeding introduced immunomodulatory factors and enhanced the pathogenesis of *R. parkeri* rickettsiosis (61). Other investigators utilized mouse infection models to determine the impacts of active tick attachment and blood feeding during *Rickettsia* infection. In one study, infesting *R. sanguineus* nymphal ticks on C3H/HeJ intradermally infected with 10^7^ R. conorii cells reduced proinflammatory responses but failed to change the histological features and bacterial loads in the lungs (62). However, in a separate study, infesting *A. maculatum* nymphal ticks on C3H/HeJ mice infected via intradermal injection of 5.5 × 10^6^ *R. parkeri* cells supported rickettsial growth, with extensive necrosis and inflammatory immune cell recruitment (63). The *R. parkeri* study utilized laboratory-reared nymphs constitutively infected with “*Candidatus* Rickettsia andeanae,” but this nonpathogenic *Rickettsia* species failed to seed infections in mice during blood feeding (63). A point mutation in the coding region of the *tlr4* gene in C3H/HeJ mice interferes with the Toll-like receptor 4 (TLR4)-mediated activation of DCs and natural killer (NK) cells important for innate rickettsial immunity and predisposes the animals to rickettsial infections (64–66). It remains unclear whether different tick species (e.g., *R. sanguineus* and *A. maculatum*) produce different classes and abundances of immunomodulatory molecules that pose various capacities to facilitate SFG *Rickettsia* survival and reduce the proinflammatory immune responses of DCs, NK cells, or other cellular components at the site of infection. Furthermore, additional studies are required to identify specific rickettsial factors that synergize with immunomodulatory factors in tick saliva, contributing to the enhanced transmission and pathogenesis of tick-borne rickettsiosis. For in-depth discussions, interested readers are directed to a recent review article highlighting the multifaceted and complex relationships between *Rickettsia* and arthropod vectors (67).

## SFG *RICKETTSIA*-ENDOTHELIAL CELL INTERACTIONS

In some cases of tick-borne rickettsioses, SFG *Rickettsia* actively replicates at tick bite sites and produces epidermal and dermal necrotic lesions that are characterized as inoculation eschars within a few days of infection. Histopathological analyses often identify vasculitis and necrotic features associated with vascular thrombosis. Numerous clinical reports have documented the presence of multiple eschars on patients infected by several SFG rickettsial agents, such as *R. parkeri*, R. philipii, (previously known as *Rickettsia* sp. strain 364D), and *R. africae* (48, 68). While most case studies (~80% of tick-borne rickettsial disease cases reported to the CDC) failed to provide information on eschars, current surveillance data suggest that eschar-associated rickettsial diseases are associated with less virulent tick-borne rickettsial agents (69). A recent investigation developed a mouse infection model (C57BL/6 mice lacking the expression of receptors for type I interferon [IFN-I] and IFN-γ) that recapitulates *R. parkeri* eschar formation upon intradermal inoculation and characterized Sca2-mediated *R. parkeri* dissemination to distal organ tissues, opening a new window of opportunity to improve our understanding of how rickettsial virulence mechanisms impact eschar formation (70).

Within the bloodstream, rickettsiae target and invade vascular endothelial cells and replicate within the cytoplasmic compartment (Fig. 1). Infections with pathogenic SFG *Rickettsia* induce increased vascular permeability associated with rickettsial replication and disruption of vascular endothelial cells with perivascular infiltration of T cells and macrophages. Progressive endothelial cell injury leads to the generation of the characteristic erythematous rash, disseminated vasculitis, cutaneous necrosis, pneumonitis, meningoencephalitis, and multiorgan failure (71). Thus, the molecular interactions between *Rickettsia* and host endothelial cells have a significant role in SFG rickettsioses. Infection of vascular endothelial cells with *Rickettsia* activates a proinflammatory state and induces cytokine and chemokine responses. Human umbilical vein endothelial cells (HUVECs) infected with R. rickettsii increased cell-associated interleukin-1α (IL-1α) production for the potential activation of IL-1 receptor 1 (IL-1R1) signaling in an intracrine and paracrine manner to coordinate the local inflammatory responses of endothelial cells and recruit professional phagocytes (72, 73). R. conorii infections in HUVECs induced cell-associated IL-1α production, triggering the secretion of IL-6 and IL-8 and the expression of adhesion molecules, including E-selectin, intercellular adhesion molecule 1 (ICAM-1), and vascular cell adhesion molecule 1 (VCAM-1) (74–76). Similarly, infections with R. rickettsii, R. conorii, and *R. africae* caused the secretion of two chemokines, IL-8 and monocyte chemoattractant protein 1 (MCP-1, also known as CCL2) from endothelial cells, implying their roles in activating and recruiting neutrophils and monocytes to the sites of infection (75, 77–79). While HUVECs exposed to heat-inactivated R. conorii generated a marked release of IL-8 and MCP-1 (CCL2) in a dose- and TLR4-dependent manner, heat-inactivated *R. africae* caused rather modest responses in HUVECs (75). In contrast to the comparable IL-8 levels in serum samples between control and African tick bite fever (ATBF) (*R. africae*) subjects, Mediterranean spotted fever (MSF) patients displayed a marked elevation of serum IL-8, potentially contributing to the distinct clinical features of MSF (75). Previous work demonstrated that IL-8 activates microvascular endothelial cells via CXCR1 and CXCR2 pathways and may contribute to vascular permeability during acute inflammation (80). It is also possible that IL-8 may inhibit and delay endothelial cell apoptosis, providing an intracellular replicative niche for *Rickettsia* (81). R. conorii infection of a mouse endothelial cell line, SVEC4-10, primed with IFN-γ or a combination of tumor necrosis factor alpha (TNF-α) and IFN-γ induced the expression of two chemokines, CXCL9 and CXCL10, known to target CXCR3 expressed on T and NK cells (important innate regulators of rickettsial infections) (82). This observation was corroborated by histopathological analysis of organ tissues collected from C3H/HeN mice infected with R. conorii (82). Human patients with confirmed cases of MSF (R. conorii) in Italy and Spain displayed pronounced increases in serum levels of CXCL10 (83). This was in part due to circulating blood cells releasing CXCL10 and additional inflammatory regulators, which promoted endothelial cells to release additional CXCL10 into the plasma (83).

Endothelial cells express CX3CL1 (fractalkine) as a transmembrane protein for the interaction with its receptor, CX3CR1, which is abundantly expressed on human innate immune cells capable of controlling early rickettsial infections, including NK cells, T cells, and monocytes/macrophages (84). Once cleaved from the surface by ADAM10 and ADAM17, soluble CX3CL1 acts as a classical chemoattractant (85, 86). *In vitro*, R. rickettsii infection of HMEC-1 cells exploited microRNA-424 to actively modulate the expression of CX3CL1. While the exact mechanisms remain unknown, *in vivo* C3H/HeN mouse infection studies performed with R. conorii confirmed that the peak expression of CX3CL1 coincides with the recruitment of macrophages during the acute phase of systemic endothelial infection (87). On the other hand, increased expression of CX3CL1 on activated endothelial cells can trigger platelet activation via CX3CR1 and enhance platelet adhesion via the glycoprotein Ibα (GPIbα) receptor (88, 89). During rickettsial infections, endothelial cell activation and subsequent injury lead to a dysregulated state of the hemostasis system (90, 91). While a minor reduction in platelet counts has often been reported for tick-borne rickettsiosis, severe coagulopathies, such as deep venous thrombosis and disseminated intravascular coagulation, have been documented for patients infected with pathogenic *Rickettsia* species, including R. rickettsii, R. conorii, R. sibirica, and *R. japonica* (92–95). Such a procoagulant potential has been documented for HUVECs infected with R. rickettsii, R. conorii, and *R. africae* (96–99). Of note, ATBF (*R. africae*) patients displayed a significantly increased level of soluble CD40 ligand (sCD40L) in serum (99). *In vitro* infection of HUVECs with *R. africae* showed a synergistic contribution of HUVECs and platelets to the elevation of sCD40L in a CX3CL1-dependent manner, potentially contributing to vascular inflammation and dysregulated hemostasis (99). These data illustrate that SFG *Rickettsia* infections of endothelial cells induce common and unique inflammatory responses. It is possible that endothelial cell responses to those species that cause mild or self-limiting tick-borne rickettsioses are beneficial and contribute to the clearance of intracellular *Rickettsia*. On the other hand, highly pathogenic SFG *Rickettsia* species may exploit host inflammatory responses to generate inappropriate local and systemic inflammatory responses, leading to limited or self-destructive hyperactive immune responses. Furthermore, it remains unknown whether specific SFG *Rickettsia* species are equipped with unique virulence factors to modulate endothelial cell responses, contributing to different clinical features. Interested readers are directed to recently published review articles that provide an excellent and detailed overview of *Rickettsia*-endothelial cell interactions (100, 101).

## SURVIVAL OF PATHOGENIC SFG *RICKETTSIA* IN PROFESSIONAL PHAGOCYTES

As rickettsiae continue to replicate and spread through the vasculature, perivascular neutrophilic and lymphohistiocytic inflammatory cells are recruited to the site of infection to prevent further dissemination of the invading bacteria (Fig. 1) (47, 102, 103). Recent investigations suggest that rickettsial survival in macrophages may determine the basis of rickettsial virulence and pathogenesis. Pathogenic SFG *Rickettsia* species, such as R. rickettsii, R. conorii, R. parkeri, R. helvetica, and R. australis, have evolved to resist bactericidal mechanisms and establish a replicative niche within the cytosolic compartments of macrophages (104–109). On the other hand, nonpathogenic R. montanensis and R. bellii fail to escape the phagolysosome and survive within THP-1 macrophages (104, 106). R. conorii replication in THP-1 macrophages induced unique proteome signatures (e.g., increased levels of proteins involved in the tricarboxylic acid cycle, oxidative phosphorylation, fatty acid β-oxidation, glutaminolysis, and mitochondrial transport) and altered metabolic and lipid catabolic pathways favoring anti-inflammatory M2 responses (110, 111). On the other hand, *R. helvetica* infection of THP-1 macrophages led to the production of the proinflammatory cytokine TNF-α (109). For *R. parkeri* survival in mouse bone marrow-derived macrophages (BMDMs), outer membrane protein B (OmpB), the most abundant and conserved protein required for the formation of protective surface- and capsule-like layers on *Rickettsia*, played a significant role in preventing the surface polyubiquitylation of OmpA and subsequent autophagy evasion (108, 112). During *R. parkeri* infection of murine BMDMs, *R. parkeri* activated the inflammasome in a caspase 1/11-dependent manner to avoid IFN-I production and the subsequent activation of interferon regulatory factor 5 (IRF5), which upregulates rickettsicidal genes encoding guanylate-binding proteins (GBPs) and inducible nitric oxide synthase (iNOS) (113). *R. australis* infections of human (peripheral blood mononuclear cell- and THP-1-derived) and mouse (BMDM) macrophages also activated inflammasome responses and induced IL-1β and IL-18 secretion in a caspase 1- and TLR4-dependent manner (107, 114). Furthermore, the activation of the inflammasome contributed to host immune control of *R. australis* in C57BL/6 mice (114). A recent study reported the role of nitric oxide in preventing protein synthesis and restricting the growth of R. rickettsii in J774 macrophage-like cells (105). These studies provide evidence that pathogenic *Rickettsia* species may exploit and evade host immune protection mechanisms and establish an intracellular niche for their survival and transmission within macrophages. Tick-borne rickettsioses present different clinical severities, ranging from life-threatening diseases to self-limiting mild cases with no complications. It remains largely unknown how individual pathogenic *Rickettsia* species employ unique or conserved virulence mechanisms to manage and foster intracellular replicative niches in macrophages, professional phagocytes equipped with an impressive armamentarium of antimicrobial mechanisms, and contribute to varying clinical severities. Many previous studies have been conducted with macrophage-like cells or murine macrophages. Although macrophage-like cells are convenient and economical, studies have demonstrated that these cells function differently in many aspects compared to primary macrophages (115). At the same time, mouse models have provided significant insights into rickettsial pathogenesis and host immunity. However, there are substantial differences between mouse and human immunology (116). Thus, such differences should be carefully considered when studying *Rickettsia*-macrophage interactions and their implications for human rickettsiosis.

## NOTABLE EMERGING SFG *RICKETTSIA* SPECIES WITH CONFIRMED OR PRESUMPTIVE HUMAN INFECTIONS

During most of the 20th century, R. rickettsii and R. conorii were considered the major tick-borne *Rickettsia* species associated with human infections (RMSF and MSF, respectively) in the Americas, Europe, and Africa (117). Over the last decades, investigators have discovered and characterized numerous novel *Rickettsia* species from ticks but considered them nonpathogenic to humans (117). However, this concept has been challenged extensively as epidemiological analyses, clinical research, and laboratory studies indicate that tick-borne rickettsioses are underdiagnosed and often associated with previously uncharacterized SFG *Rickettsia* species (118). As discussed here and elsewhere, recent investigations describe that emerging SFG *Rickettsia* species are capable of infecting cells in the salivary glands and midguts of ticks, display moderate virulence in *in vitro* and *in vivo* infection models, produce mild-to-moderate clinical disease in patients, and elicit inflammatory and pathogen-specific immune responses after tick transmission. Among many emerging SFG *Rickettsia* species, five such examples are selected and reviewed in detail below to illustrate how recent studies provided insights into understanding *Rickettsia*-host-tick interactions (additional emerging SFG *Rickettsia* species are reviewed in references 118 and 119).

### R. parkeri.

*R. parkeri* was initially isolated from *A. maculatum* ticks collected from cows in Texas in 1939 and characterized for mild febrile disease with edema and reddening of the scrotum in guinea pigs (120). After >60 years of speculation, the pathogenicity of *R. parkeri* was confirmed with a patient in Virginia presenting relatively mild febrile illness accompanied by multiple eschars and a maculopapular eruption (121). Similar to other tick-borne rickettsial infections, *R. parkeri*-infected patients display a combination of nonspecific clinical symptoms (e.g., fever, headache, malaise, and myalgia) and characteristic eschars at the inoculation site of tick attachment (122–124). Some *R. parkeri*-infected patients required hospitalization, but no case fatalities have been reported for *R. parkeri* infections (125). Bioinformatics determined the syntenic organization of the three *R. parkeri* genome sequences with differences in genome sizes (Atlantic Rainforest, 1.35 Mbp; Black Gap, 1.33 Mbp; Portsmouth, 1.30 Mbp) and gene rearrangements, partly due to the presence or absence of *tra* genes (126). *R. parkeri* is associated with several *Amblyomma* ticks (*A. maculatum* as the main vector) in the Americas (127–129). Over the last decades, many tick species, including *A. maculatum*, have expanded their ranges, seeding new habitats and potential hot spots for *R. parkeri* rickettsiosis (130, 131). The distinct geographical distributions of *Amblyomma* species may have contributed to the phylogenetic differentiation and evolutionary adaptation of multiple *R. parkeri* species (132). However, *R. parkeri* infections caused by various *Amblyomma* vectors do not cause significant differences in clinical outcomes (133).

The identification of genes required for the rickettsial intracellular life cycle is an essential step toward understanding the molecular basis of tick-borne rickettsiosis (134). Furthermore, information on the essential molecular mechanisms will assist in deducing vaccine antigens and therapeutic drug targets. Over the last decades, several genetic tools have been developed to create bacterial variants that carry insertional, deletional, or point mutations and to study the consequences of mutations using *in vitro* and *in vivo* infection models. However, the genetic intractability of obligate intracellular bacteria, including *Rickettsia*, has set a significant barrier to genetic tools readily available for free-living bacteria. Despite numerous technical limitations, recent work described the stable transformation of *Rickettsia* with plasmid DNA in the presence of antibiotic selection and established random transposon mutagenesis systems for *R. prowazekii*, R. rickettsii, *R. parkeri*, and R. conorii (2, 135–137). Using *R. parkeri* insertional mutants, Welch and colleagues demonstrated that (i) Sca2 and RickA mediate actin-based motility in a time-dependent manner in tissue culture cells, (ii) Sca2 is required for *R. parkeri* pathogenesis and dissemination from the inoculation site to internal organs in *Ifnar1*^−/−^ *Ifngr1*^−/−^ mice, (iii) Sca4 associates with vinculin and mediates the cell-to-cell spread of *R. parkeri*, and (iv) OmpB (Sca5) blocks the ubiquitylation of rickettsial surface antigens to promote autophagy evasion in immune cells and contributes to eschar formation in *Ifnar1*^−/−^ *Ifngr1*^−/−^ mice (70, 108, 138–140). These studies demonstrate the usefulness of transposon mutagenesis for the study of rickettsial pathogenesis. However, the obstacles to creating a saturated *Rickettsia* mutant library remain due to low transformation efficiency and the use of long-term cultivation to recover, isolate, and determine the genetic lesions of clonal variants in the tissue culture system.

### R. africae.

Genetic analysis of *R. africae* identified a circular chromosome (1.28 Mbp; 32.4% G+C content) and a circular pRA plasmid (12.3 kbp; 33.4% G+C content), with 1,260 chromosomal and 11 plasmid open reading frames (ORFs) predicted (20). The *R. africae* and R. conorii chromosome sequences displayed almost perfect collinearity except for an ~88.5-kbp inversion that harbors *tra* gene orthologs encoding components of a T4SS for conjugal DNA transfer present in *R. africae* but not R. conorii (20). The contributions of the T4SS to *R. africae* (or SFG *Rickettsia*) pathogenesis, survival in mammalian and arthropod hosts, and transmission remain unclear (27). *R. africae* causes ATBF and infects multiple species of *Amblyomma* (A. hebraeum and A. variegatum as the main tick vectors), *Hyalomma*, and *Rhipicephalus* ticks in areas of endemicity in sub-Saharan Africa (141–144). Transstadial and transovarial transmissions of *R. africae* have been reported for *A. hebraeum* (145, 146). A recent PCR analysis identified that most *Amblyomma* ticks (up to 100%) collected from cattle in south and central Mozambique are infected with *R. africae* (143). Similarly, *R. africae* infection was prevalent in A. variegatum ticks (87%) on cattle in Madagascar (147). In the coastal region of the Eastern Cape, PCR amplification and sequencing analysis of the *gltA*, *ompA*, *ompB*, *sca4*, and 17kDa genes identified the presence of *R. africae* in *A. hebraeum* (63% adults and 62% nymphs) and blood from cattle (22%) (144). A previous serosurvey of cattle in Zimbabwe identified antibodies cross-reactive to *R. africae*, implying that cattle may play an important role in *R. africae* maintenance in Africa (148). In contrast, a recent study determined that only a small number of A. variegatum, Rhipicephalus decoloratus, and R. evertsi *mimeticus* ticks collected from domestic cattle in Angola were infected with *R. africae* and found no *R. africae* DNA in bovine blood (149). *R. africae* infections of *A. hebraeum* ticks on goats in Mpumalanga Province (eastern South Africa) and A. variegatum ticks on goats, sheep, and cattle in Kenya suggest that other ruminants may serve as alternative hosts for *R. africae* transmission and amplification in nature (142, 150). Additional molecular detection and serosurvey studies are necessary to define mammalian hosts for *R. africae* and their implications for human infections in different geographical areas of Africa.

Most reported cases of ATBF are from international travelers in African countries. In fact, *R. africae* is the second most frequent etiological agent of febrile diseases, after malaria, among tourists returning from southern Africa with a history of travel to grasslands and game parks (estimated infection rate of ~5%) (151, 152). This is partly due to the lack of scientific and public health infrastructures for prompt molecular diagnosis and disease surveillance in these areas of endemicity. Previous seroprevalence studies reported high rates of antibody cross-reactivity to SFG *Rickettsia* in many African populations (153–155). In studies performed in Cameroon, seroprevalence studies identified rates of positivity for antibodies reactive with *R. africae* of 27 to 32%, suggesting that ATBF is common in African countries of endemicity (156, 157). Recent environmental changes, international travel, and shipments contribute to the expansion and invasion of ticks into new geographical habitats. The recent identification of *R. africae*-infected A. variegatum on cattle in Corsica, France, and on sheep in Sardinia, Italy (two islands located in the Mediterranean Sea), illustrates the importance of sustained surveillance for the expansion of ticks and the occurrence of associated tick-borne pathogens of veterinary and medical significance (158, 159). Infections with *R. africae* produce nonspecific flu-like clinical signs, including headache, fever, eschars, rash, lymphadenopathy, myalgia, chills, malaise, and arthralgia. Patients often report multiple eschars formed at the tick bite sites on the lower extremities, as the main tick vectors, A. variegatum and *A. hebraeum*, display aggressive host-seeking behavior, and patients are often bitten by multiple infected ticks simultaneously (152). While R. conorii causes MSF in similar geographical areas of Africa, MSF is often associated with a history of contact with *R. sanguineus* and severe clinical outcomes compared to ATBF (160). Most *R. africae* infections cause mild and self-limiting disease, but some severe manifestations, such as cardiomyopathy, neuropathy, cellulitis, retinitis, and chronic fatigue, have been reported in elderly patients (161–163).

### R. heilongjiangensis.

*R. heilongjiangensis*, first isolated in 1982 from Dysmicoccus sylvarum ticks in Suifenhe, a city in the Heilongjiang Province of China, is comprised of a 1.28-Mbp (32.3% G+C content) circular chromosome and no plasmid DNA (164, 165). Comparative genomics analysis suggests that *R. heilongjiangensis* is closely related to *R. japonica* and causes Far-Eastern spotted fever transmitted by Haemaphysalis japonica, H. concinna, H. longicornis, and D. sylvarum in China, Siberia, the Russian Far East, and Japan (25, 39, 166–170). Clinical evaluations revealed common symptoms associated with tick-borne rickettsiosis without significant complications and mortality (39, 166). Patients with a history of a tick bite developed nonspecific clinical symptoms, including sudden onset of fever, chills, and headache, along with maculopapular rash, eschar at the tick bite sites, subcutaneous lymphangitis, and regional lymphadenopathy (39, 166). Mouse infection models have been explored to study the pathological potential of *R. heilongjiangensis*. Intravenous injection of 10^5^ viable *R. heilongjiangensis* cells induced hematogenous dissemination, interstitial pneumonia, systemic infection, and multifocal inflammatory lesions with immune cells in multiple organ tissues but failed to cause lethal disease in BALB/c mice (171). Other studies demonstrate that high infectious doses (up to 10^8^ cells) of *R. heilongjiangensis* are required to induce nonlethal acute disease when intraperitoneally injected into C3H/HeN or C57BL/6 mice, corroborating clinical observations of mild cases in patients infected with *R. heilongjiangensis* (171–174).

### R. honei.

*R. honei* causes Flinders Island spotted fever (FISF) in patients bitten by the reptile tick B. hydrosauri in Australia and Thai tick typhus in those exposed to Ixodes granulatus bites in Thailand (175–178). Whole-genome sequencing of *R. honei* strain RB^T^ revealed a 1.26-Mbp chromosomal DNA (32.4% G+C content) predicted to harbor 1,284 genes closely related to the genes present in R. rickettsii, R. conorii, and R. slovaca and no plasmid DNA (179). In *B. hydrosauri*, *R. honei* was present in multiple organs, including salivary glands and oocytes, potentially facilitating transstadial and transovarial transmission (178). However, a recent tick survey failed to detect by PCR the presence of *R. honei* in *B. hydrosauri* ticks harvested from skinks (Tiliqua rugosa) in southern Australia, where confirmed cases of FISF have been reported (180). Thus, active surveillance of ticks present in areas where FISF is endemic is needed to reveal the primary reservoirs of *R. honei* among different geographical regions (181). *R. honei* subsp. *marmionii* infects H. novaeguineae ticks and causes Australian spotted fever (ASF) (182, 183). Clinical symptoms of FISF and ASF are mild and similar to those of other tick-borne rickettsioses and include fever, headache, arthralgia, myalgia, maculopapular/petechial rash, and eschar formation in some cases (176, 177, 182, 183). Of note, atypical chronic and severe infections have been reported in some patients (182, 183). A recent case report described the first probable death of a middle-aged patient infected with *R. honei*, potentially due to delays in diagnosis and doxycycline treatment (184). Cases of FISF and ASF have also been confirmed in a U.S. traveler returning from India and a patient in Nepal, suggesting the broader existence of *R. honei* in multiple geographical areas (185, 186). While previous work described moderate pathogenicity of *R. honei* in guinea pigs and gerbils, additional studies are required to characterize the pathologies associated with *R. honei* infections in animal infection models that reflect FISF and ASF, compare immunopathological features to those of other SFG *Rickettsia* species, and study their role in tick infection and transmission in reptile populations (187).

### R. amblyommatis.

The genome of R. amblyommatis GAT-30V consists of a single chromosome (1.41 Mbp; 32.4% G+C content) and three circular pRM plasmids (32 kbp, 18 kbp, and 23 kbp). Compared to pathogenic SFG *Rickettsia* species (R. rickettsii and R. conorii), the chromosomal DNA sequence of *R. amblyommatis* displayed high degrees of sequence identity, multiple genetic rearrangements, and regions undergoing gene decay (34). Bioinformatics identified putative virulence genes present with 86 to 95% amino acid identity, but their biological roles in *R. amblyommatis* pathogenesis, transmission, and replication in ticks remain unresolved (30). *R. amblyommatis* frequently infects Amblyomma americanum ticks (up to 64%) (188). In recent years, A. americanum has rapidly expanded its habitats into the Northeast and Midwest and has become the dominant tick species, contributing to an increased number of patients with tick-borne febrile illnesses of an unknown etiology (189–191). On the other hand, the current prevalence of R. rickettsii (RMSF) in D. variabilis is estimated to be less than 1% (188). Seroprevalence studies suggest that domestic and wild small mammals (dogs and cats) may contribute to *R. amblyommatis* transmission and amplification, with humans as an accidental host (192, 193). However, a definitive mammalian host with systemic *R. amblyommatis* infection as a source of *R. amblyommatis* infection of ticks has not been identified and may vary by geographical location. Nonetheless, *A. americanum* ticks display nondiscriminative and aggressive biting behavior and pose a significant public health concern as they can frequently cause *R. amblyommatis* rickettsiosis and other tick-borne diseases such as ehrlichiosis, Southern tick-associated rash illness, and tularemia (194). In addition, *Amblyomma* tick bites are associated with an unusual life-threatening allergic reaction to oligosaccharide galactose-α-1,3-galactose (α-Gal), also known as meat allergy, but the underlying cause remains unknown (195–197).

A previous case report described a patient bitten by an *A. americanum* tick who developed a macular rash at the tick bite site and had a positive PCR test for *R. amblyommatis* and the absence of *Borrelia*, *Ehrlichia*, *Anaplasma*, *Babesia*, *Bartonella*, and other pathogenic *Rickettsia* species (198). Other studies reported that patients diagnosed with probable RMSF developed *R. amblyommatis*-specific antibodies and presented typical clinical manifestations of tick-borne rickettsioses such as fever, headache, and myalgia, suggesting that *R. amblyommatis* is a presumptive etiological agent for mild rickettsiosis (199–201). Recent and previous investigations studied the virulence potential of *R. amblyommatis* using *in vitro* and *in vivo* infection model systems and largely corroborated the current clinical evidence that *R. amblyommatis* may cause self-limiting mild tick-borne rickettsiosis. *In vitro* tissue culture infection of HMEC-1 cells showed that *R. amblyommatis* displays defective host cell attachment and replicates within host endothelial cells at a lower rate than R. conorii (34). Guinea pigs infected with intradermal and intraperitoneal injections of 10^6^ cells of *R. amblyommatis* North Texas (isolated from *A. americanum*) did not show any clinical illness but seroconverted by 14 days postinfection (202). In a separate study, intraperitoneal injection of 4 × 10^6^ cells of *R. amblyommatis* 9-CC-3-1 (isolated from Amblyomma cajennense) caused vascular inflammation in guinea pigs (203). Of note, both studies demonstrated that guinea pigs infected with *R. amblyommatis* generated cross-reactive immunity that protected the animals against a lethal challenge with R. rickettsii (202, 203). A recent comparative analysis of disease severity in guinea pigs infected with R. rickettsii or *R. amblyommatis* determined that *R. amblyommatis* infections caused much milder clinical manifestations than R. rickettsii (204). Similarly, significantly higher infectious doses of *R. amblyommatis* were required to cause sublethal and lethal diseases in C3H/HeN mice than in those infected with R. conorii (34). While these studies corroborate clinical evidence that *R. amblyommatis* may cause mild tick-borne rickettsiosis in some patients, future studies need to utilize tissue culture-based and molecular-based diagnostic approaches to evaluate skin biopsy samples from patients bitten by *A. americanum* ticks, establish a causal relationship for *R. amblyommatis* rickettsiosis, and perform comparative analyses to determine the genetic diversities of *R. amblyommatis* strains present in *A. americanum* ticks in traditional and new habitats in the Americas.

## CONCLUDING REMARKS

Several advances have been made within the last decade toward understanding basic *Rickettsia* biology (e.g., genomics, pathogenicity, and vector competence and transmission) and developing molecular tools for *Rickettsia*. Yet some deficiencies (e.g., transmission mechanisms, epidemiology, species diversity, and tick biology) continue to hinder investigative advances for this universal emerging pathogen, highlighting significant research opportunities for discovering novel molecular mechanisms associated with the obligate intracellular life cycle of SFG *Rickettsia* species.
